# Flexible Mid-infrared Photonic Circuits for Real-time and Label-Free Hydroxyl Compound Detection

**DOI:** 10.1038/s41598-019-39062-z

**Published:** 2019-03-11

**Authors:** Tiening Jin, Hao-Yu Greg Lin, Tom Tiwald, Pao Tai Lin

**Affiliations:** 1 Department of Electrical and Computer Engineering, Texas A&M University, College Station, Texas 77843 United States; 2 Department of Materials Science and Engineering, Texas A&M University, College Station, Texas 77843 United States; 3 Center for Remote Health Technologies and Systems, Texas A&M University, College Station, Texas 77843 United States; 4 Center for Nanoscale Systems, Harvard University, 11 Oxford Street, Cambridge, Massachusetts 02138 United States; 5 J. A. Woollam Co., Inc., Lincoln, NE 68508 USA

## Abstract

Chip-scale chemical detections were demonstrated by mid-Infrared (mid-IR) integrated optics made by aluminum nitride (AlN) waveguides on flexible borosilicate templates. The AlN film was deposited using sputtering at room temperature, and it exhibited a broad infrared transmittance up to λ = 9 µm. The AlN waveguide profile was created by microelectronic fabrication processes. The sensor is bendable because it has a thickness less than 30 µm that significantly decreases the strain. A bright fundamental mode was obtained at λ = 2.50–2.65 µm without mode distortion or scattering observed. By spectrum scanning at the -OH absorption band, the waveguide sensor was able to identify different hydroxyl compounds, such as water, methanol, and ethanol, and the concentrations of their mixtures. Real-time methanol monitoring was achieved by reading the intensity change of the waveguide mode at λ = 2.65 μm, which overlap with the stretch absorption of the hydroxyl bond. Due to the advantages of mechanical flexibility and broad mid-IR transparency, the AlN chemical sensor will enable microphotonic devices for wearables and remote biomedical and environmental detection.

## Introduction

Flexible integrated photonics has attracted significant attention because it enables applications including portable imaging, optical links, strain sensing, and wearable photonic textiles^1–11^. Flexible semiconductor photonic devices, such us light-emitting diodes, photodetectors, and Fano reflectors have been utilized in free-space-coupled optical components^12–15^. In parallel, planar photonic devices, such as microscale optical waveguides and micro-resonators, provide additional advantages when compared with their free-space counterparts due to their capability to program optical information in a chip, and ability to join with current microelectronics and wireless components. Nevertheless, flexible photonics operating in the mid-IR spectrum regime have not been fully developed because the majority of them were made by organic polymers that are opaque in the mid-IR region. Furthermore, these polymeric substrates decompose and deform under high temperatures and degrade when exposed to organic solvents, limiting their applications in wearable sensing and severe environmental monitoring.

To overcome these difficulties, an ultra-thin borosilicate sheet was utilized as the flexible substrate to support the bendable photonic circuits. Bulk borosilicate is considered hard and brittle. However, a thin borosilicate sheet can be bent without cracking because the strain from bending is inversely proportional to the thickness of the template. For sensing applications, the borosilicate sheet offers the following advantages: (i) wide spectral transmission, (ii) chemical and thermal stability, and (iii) complementary metal-oxide-semiconductor (COMS) fabrication compatibility. In detail, i. borosilicate is transparent over the near-IR and the mid-IR until λ = 3.4 µm, which extensively covers the characteristic absorptions of numerous functional groups. These include -NH, -CH, -OH, etc., thus allowing borosilicate for label-free chemical detections^16–18^. Furthermore, for ii. borosilicate has high thermal and chemical resistance, so it can perform chemical and toxic detection in extreme environments. iii. The borosilicate sheet is able to be integrated with semiconductor materials and microelectronic processes. Therefore, borosilicate sheets have been widely applied in manufacturing, such as in the production of microelectronics and optical devices.

However, an optical waveguide consists of two components: the waveguide cladding made by a lower refractive index material like borosilicate and the waveguide core with a higher index material^19^. AlN is an interesting high index material for photonic circuits due to its broad transmission window from ultraviolet to mid-IR at λ = 10 μm^20,21^. Meanwhile, high-quality AlN thin films have been deposited on numerous microelectronic templates, such Si, SiO_2_, or sapphire wafers by atomic layer deposition (ALD), chemical vapor deposition (CVD), or sputtering^22–25^. Furthermore, AlN reveals high optical nonlinearities ready to be applied in nonlinear photonic devices, including frequency up- and down- conversions^26^.

By means of the mid-IR and mechanical properties of AlN and borosilicate, the AlN waveguides with the flexible borosilicate were joined to build bendable photonic devices. Finite difference method (FDM) was applied to simulate the waveguide modes and their sensing effects. The AlN thin films were prepared by room temperature direct current (DC) sputtering. The optical properties of the deposited AlN were measured by infrared variable angle spectroscopic ellipsometry (IR-VASE). The AlN ridge waveguides were then developed on the borosilicate sheet through the CMOS processes. The waveguide mode profiles were recorded and examined at λ = 2.50–2.65 μm. To assess its label-free and real-time sensing performance, analytes including methanol, ethanol, water, and mixtures of these were monitored by measuring their respective -OH stretch characteristic absorptions. Therefore, we showed that our flexible AlN-on-borosilicate device enabled chip-scale, label-free, and *in situ* chemical detection.

## Experimental Methods

**Figure 1:**
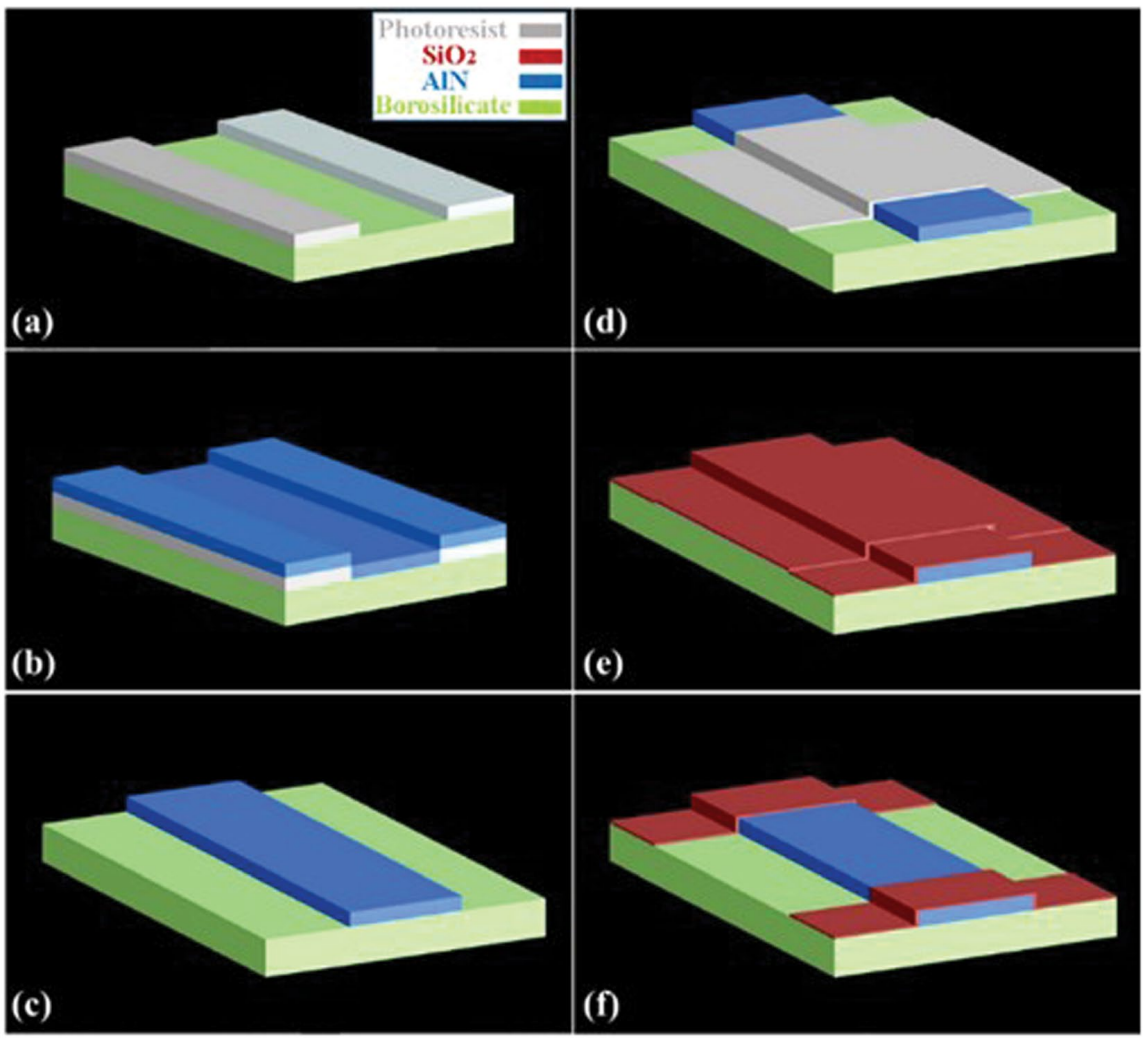
The fabrication process of the device. (**a**) The waveguide structure was patterned on the borosilicate template by photolithography. (**b**) The AlN layer was deposited on the template through DC sputtering. (**c**) The ridge waveguide structure was developed after lift-off process. (**d**) Second lithography defined the sensing aperture. (**e**) SiO_2_ was deposited as the top cladding layer. (**f**) The sensing aperture formed after the second lift-off process.

Figure 1 illustrates the device fabrication process. At first, the waveguide structure was defined on a 25 µm thick borosilicate template by photolithography. An AlN film was deposited on the borosilicate sheet by DC magnetron sputtering. Ar was pre-injected inside the chamber to clean the surface of the Al target. The working pressure was 10 mTorr and the sputtering power was 1 kW. The deposition rate was 1 µm/hour when the borosilicate template was 15 cm away from the Al target. During the lift-off step, the photoresist and the AlN on it were removed so that 2 µm tall AlN waveguides were left on the borosilicate template. At last, a 250 nm thick SiO_2_ top-cladding layer with an open aperture in the waveguide center was created by RF sputtering.

**Figure 2:**
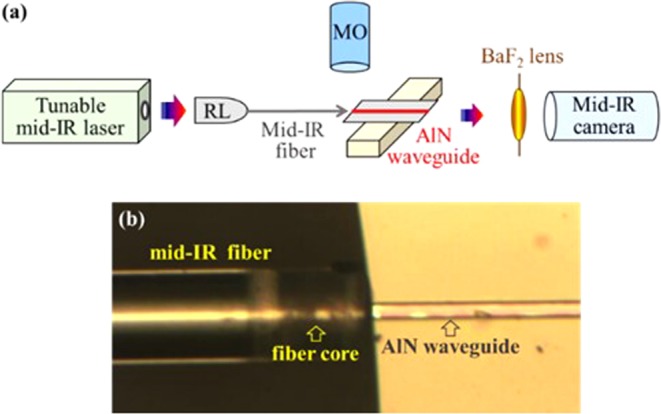
(**a**) The schematic of the experimental set-up to test the waveguide and the sensor. (**b**) The optical image shows the fine alignment between the mid-IR optical fiber and the AlN waveguide.

Figure 2a illustrates the test station built to measure the mid-IR device. The mid-IR light from a 10 ns pulsed laser with a 150 mW average power was butt-coupled to the AlN waveguide through a lens and a fluoride single mode fiber. Figure 2b shows the repositioning of the fiber and the AlN waveguide observed by a microscope. The light signals from the waveguide end facet were collected by a BaF_2_ lens and then captured by a mid-IR camera, cooled with liquid nitrogen. The flexible AlN waveguide device was placed on a flat and then a curved sample holder to measure its performance with and without mechanical bending. During the chemical sensing test, a 0.5 mL chemical was dropped on the 0.5 cm^2^ device so that the waveguides were completely wetted by the analyte. To perform blind testing, analytes consisting of water, ethanol, or methanol were transferred into numerous beakers without labelling. These unknown analytes were dropped on the device, and then the waveguide modes were measured at λ = 2.50–2.65 µm to identify their chemical compositions. The experiments were performed at 25 °C.

## Results and Discussion

**Figure 3:**
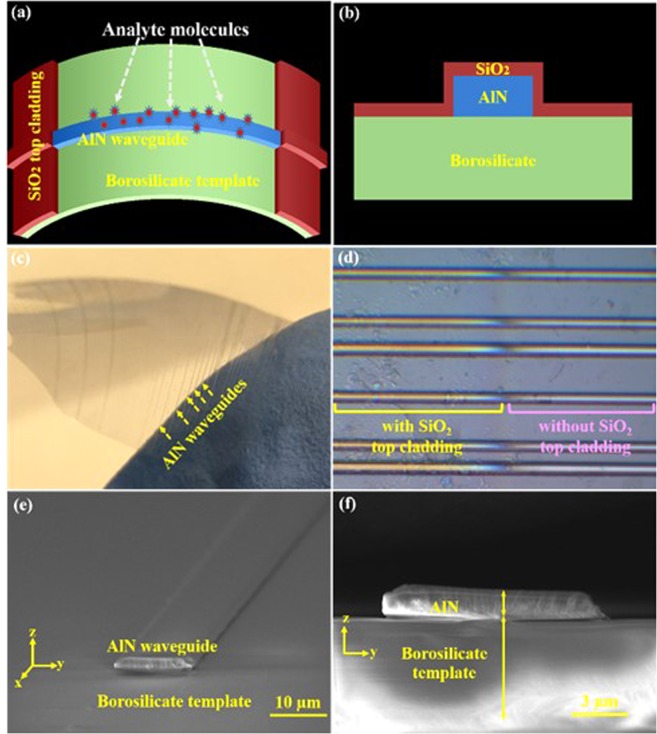
The schematics of the bendable AlN-on-borosilicate waveguide displayed from the (**a**) top and (**b**) cross-section. The center is exposed to its surroundings for sensing applications. Meanwhile, the SiO_2_ cladding covers the waveguide ends and anchors the waveguide onto the borosilicate template. (**c**) The created ultra-thin and flexible waveguides. The waveguide array is indicated by the blue arrows. (**d**) The enlarged optical image of the AlN waveguides. The left section of the waveguide array is covered by the SiO_2_ top cladding. The right section remains exposed to the environment for sensing applications. The (**e**) top and (**f**) cross-sectional SEM images of a 2 μm × 10 μm AlN waveguide. Smooth waveguide surfaces and a sharp interface between the waveguide and the borosilicate template are clearly resolved.

Figure 3 illustrates the design and structure of the flexible AlN waveguide on the borosilicate template. In Fig. 3a, the SiO_2_ top cladding was placed on the ends of the waveguide to anchor the edges of the waveguide onto the template. Meanwhile, the center of the waveguide was open so that it was exposed to the surrounding chemicals for sensing applications. The chemicals next to the waveguide absorb its evanescent field and attenuate the intensity of the waveguide mode. Therefore, the constituents and the concentrations of chemical mixtures can be identified *in situ* by examining the spectral change of the waveguide light. Figure 3b shows the cross-sectional structure of the AlN waveguide between the top SiO_2_ layer and the lower borosilicate layer. Figure 3c illustrates the fabricated bendable waveguides. No detachment or discontinued sections were observed when the device was bended. This verified that the AlN waveguides were firmly joined with the borosilicate sheet and were able to stand high stress. Figure 3d displays the enlarged image of the device. In the left section of the image, a thin SiO_2_ cladding covers the AlN waveguide and prevents it from detaching during bending or stretching. In the right section of the image, the waveguide is open to the environment for detection applications. The structure detail of the waveguides was further inspected by scanning electron microscopy (SEM). Figure 3e,f display the top and the cross-sectional SEM images of a 2 μm high and 10 μm wide AlN waveguide. The waveguide had a clear ridge profile without defects observed along the edges or on its surface. The smooth waveguide surface prevents propagation loss due to scattering, which is a critical factor to perform accurate waveguide sensing. In addition, the interface between the AlN waveguide and under-cladding borosilicate template is well-resolved. No depletion damage was found on the device surfaces or the interface since the AlN waveguides were prepared by the lift-off process instead of applying an aggressive etching process.

**Figure 4:**
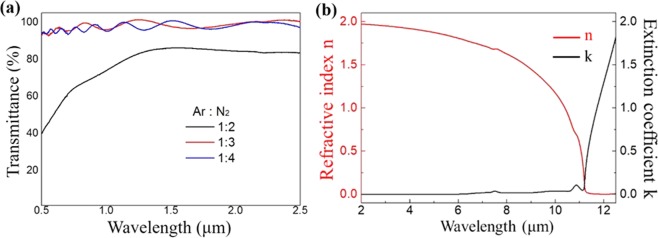
(**a**) Vis-NIR spectrum of the AlN films prepared by DC sputtering at different Ar:N_2_ ratios. The AlN film is transparent when the Ar:N_2_ ratio reached 1:3, indicating that there was no metallic Al residue left in the film. (**b**) The **n** and **k** plots of the AlN thin film from IR-VASE measurement. The n has low dispersion up to λ = 9 μm, and negligible absorption is found before λ = 10 μm. The increase of k after λ = 10 μm was due to the absorption of the Al-N bond.

The material property of the deposited films was characterized by a Vis-NIR spectrometer. As shown in Fig. 4a, the film prepared at N_2_/Ar = 2:1 was not transparent in the visible or infrared regions due to the unreacted Al metal residues in the AlN film. On the other hand, the two AlN films that were prepared at higher N_2_/Ar ratios were fully transparent from λ = 0.5 to 2.5 µm because all of the sputtered Al atoms were reacted with the nitrogen molecules and no metallic Al was left in the film. The optical quality, including the transparency, of our AlN film is comparable to others prepared by high temperature sputtering. The optical constants of the deposited AlN, including its index of refraction n and extinction coefficient (imaginary refractive index) k, were further characterized by IR-VASE between λ = 2 µm and 13 µm. As shown in Fig. 4b, the n decreased slowly from 1.9 at λ = 2 μm to 1.6 at λ = 9 μm until a dispersion was observed after λ = 10 μm. The relatively low k observed before λ = 10 μm reveals its potential for application in broadband mid-IR photonic circuits. The absorption after λ = 10 µm was caused by the Al-N stretching absorption that corresponds to the longitudinal optical (LO) mode and the transverse optical (TO) E1 mode of the Al-N bond^27,28^.

**Figure 5:**
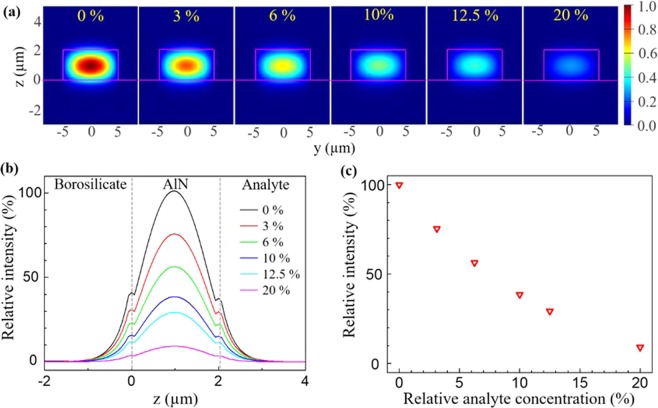
(**a**) The calculated mode images of an AlN-on-borosilicate waveguide when it was exposed to an analyte containing a mid-IR absorptive chemical. The concentrations of 0%, 3%, 6%, 10%, 12.5%, and 20% were utilized in the modeling. The waveguide mode gradually vanished when the concentration increased. (**b**) The mode intensity profiles along the z-axis at y = 0 μm. Both the guided light (0 μm < z < 2 μm) and the evanescent field decrease when the chemical concentration increased. (**c**) The plot of waveguide mode intensity vs. analyte concentration. The relative mode intensity dropped from 1 to 0.1 as the concentration increased from 0% to 20%.

The waveguide sensing performance was numerically studied by the two-dimensional finite element method (FEM). The optical modes of the AlN waveguide were calculated when it was exposed to a mid-IR absorptive chemical. The structure parameters utilized in the modelling were obtained from the SEM images shown in Fig. 3f, where the AlN waveguide had a 2 μm x 10 μm structure, and the refractive index of AlN and borosilicate were 1.97 and 1.46, respectively. The waveguide mode was excited at λ = 2.65 μm since it overlapped with the absorption band of -OH. The transverse magnetic (TM) polarization was utilized because the AlN ridge waveguide had a large y:z aspect ratio of 5:1 that created a strong evanescent field along the z-direction. A strong evanescent field is essential for achieving high sensitivity because the detection ability of the sensor improved when the interaction between the field and the chemicals approaching the waveguide increased. Figure 5a displays calculated mode images when the waveguide was surrounded by analytes with different concentrations of a mid-IR absorptive chemical. Here, the k of the analyte is proportional to the chemicals’ concentration. A fundamental mode with an elliptical intensity distribution was found inside the AlN waveguide, and its evanescent field extended into both the surrounding chemicals (z > 2 µm) and the borosilicate layer (z < 0 µm). The waveguide mode faded quickly as the chemical concentration increased because the evanescent wave was considerably absorbed by the chemical moving close to the waveguide. To analyze the waveguide modes when mid-IR absorption increases, Fig. 5b displays the intensity profiles of the TM polarization modes along the z-axis as the concentration changes. The intensities of the guided wave (0 < z < 2 µm) and the evanescent wave (z > 2 µm and z < 0 µm) both decreased drastically when the concentration of the absorptive chemical increased. Yet, the waveguide mode remained a fundamental mode regardless of the concentration. The invariance of the mode profile is critical to achieve accurate waveguide sensing since the formation of modes in higher orders changes the mode structure and the evanescent wave intensity that consequently cause signal variation during the sensing measurements. Figure 5c plots the waveguide mode intensity when the analyte concentration was consistently increased from 0% to 20%. The mode intensity decreased monotonically as the chemical concentration increased. The results show that the mid-IR waveguide is able to perform accurate concentration analysis by reading the intensity variations of the waveguide mode.

**Figure 6:**
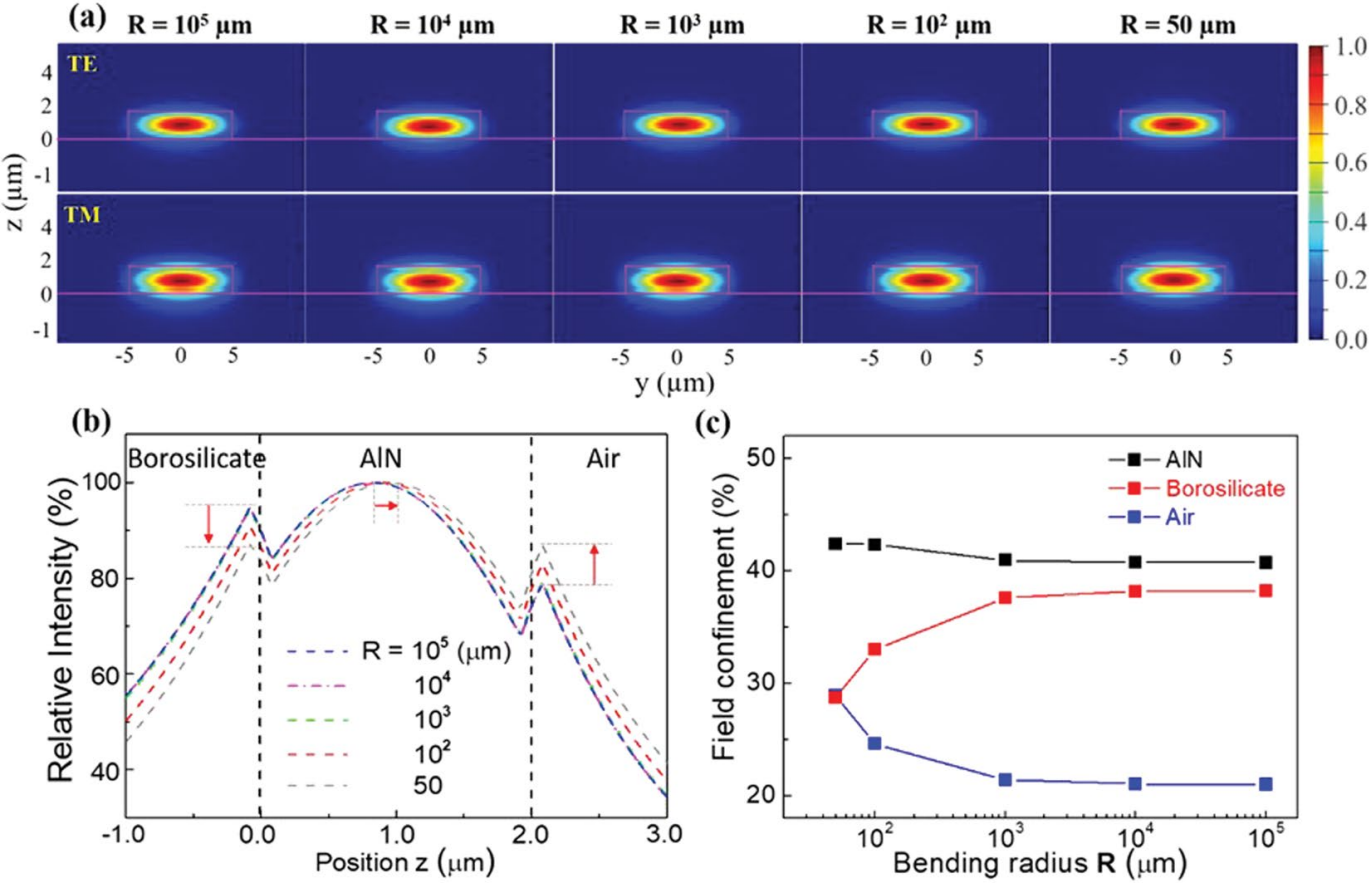
(**a**) The FEM calculated waveguide modes of a 2 µm tall AlN-on-borosilicate waveguide at various bending radii R. The first and second rows represent the TE and TM polarized modes, respectively. The wavelength is at **λ** = 2.65 µm. No shifting was found in TE mode as R changed. The TM mode slightly shifted toward the air as the bending deformation was applied. (**b**) The intensity distribution along the z-axis for the TM polarized modes when R increased from 10^5^ to 50 µm. The evanescent field in the borosilicate template decreased while the other evanescent field in air region increased. (**c**) The TM optical field confinement inside the AlN waveguide, the upper air region, and the lower borosilicate cladding. Though the evanescent fields varied at different R, the field guided in the AlN layer remained almost the same.

The bending effect of the flexible device was evaluated by calculating the waveguide mode profile at various bending radii R. Figure 6a displays the modes of the 2 µm thin AlN waveguide when the flexible device was warped at R = 10^5^, 10^4^, 10^3^, 10^2^, and 50 µm, respectively. For a transverse electric (TE) polarization, the fundamental mode revealed the same elliptical profile when the R was changed considerably from 10^5^ to 50 µm. This indicates that the structure deformation had a negligible impact on the waveguide properties since the majority of the optical field was still confined inside the high index AlN layer. For a TM polarization, the mode slightly shifted toward the air when the bending deformation was applied. Figure 6b illustrates the TM polarized optical fields calculated at different R. The center of the mode moved toward the lower refractive index of the air by only 70 nm. Figure 6c plots the TM optical field confinement factors inside the AlN waveguide, the top air, and the lower borosilicate cladding layer. The light field distribution into those three layers were stable and consistent when the R was larger than 10^3^ µm, and the wave confined inside the AlN layer remained consistent at 41%. The results indicate that the light mode is able to tolerate intense structure deformation.

**Figure 7:**
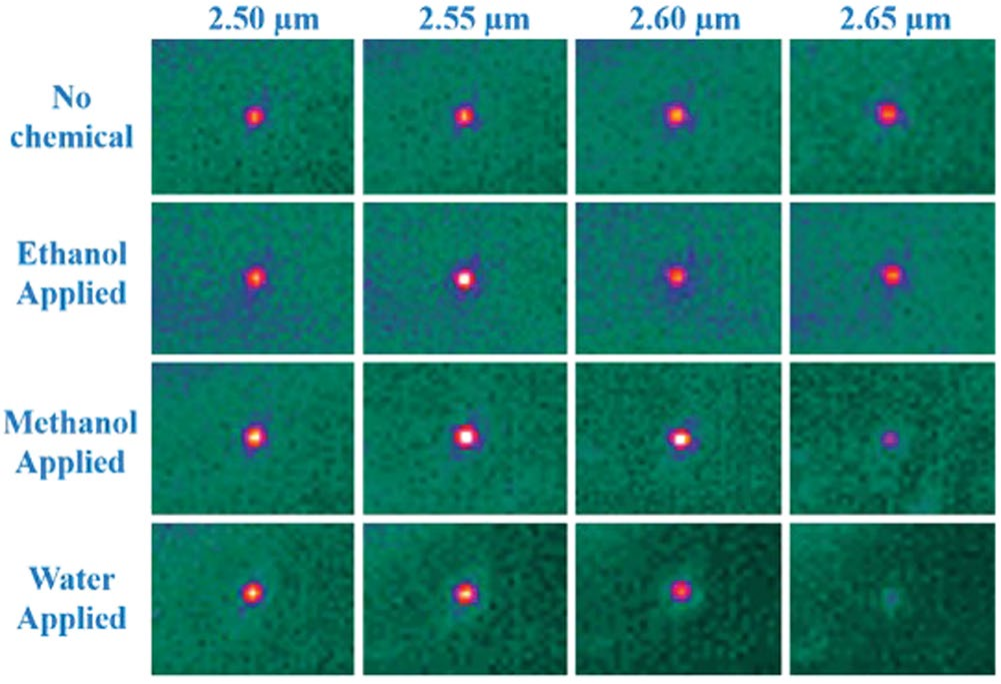
The waveguide mode images captured between λ = 2.50 and 2.65 µm when it was covered by different hydroxyl chemicals, including methanol, ethanol, and water. The absorption of water raised at λ = 2.60 µm and the mode vanished at λ = 2.65 µm. For the methanol wetted waveguide, the mode became dim until λ = 2.65 µm. The mode of the ethanol wetted waveguide remained bright, indicating that ethanol is transparent at λ = 2.50–2.65 µm.

To evaluate the sensing performance of the AlN flexible waveguides, chemicals containing a hydroxyl group were chosen as analytes due to their strong -OH characteristic absorption between λ = 2.6 and 3.3 µm. The TM mode light was utilized since it had a strong evanescent field, enabling sensitive chemical detection. The wavelength of the light was tuned between λ = 2.50 and 2.65 µm, where the -OH absorption rose and the AlN waveguide was transparent. The image of the waveguide mode was captured with and without the presence of the analytes on the waveguide. Figure 7 shows a clear waveguide mode between λ = 2.50 and 2.65 µm when no chemical was applied. The same waveguide mode profile was observed when the flexible waveguide devices were mechanically bended. In addition, the mode profiles remained the same at different wavelengths without any scattering. No distortion was observed in the captured modes, indicating that the waveguides had a clear sidewall and a sharp interface between the AlN and borosilicate layers. The high refractive index difference of the AlN and the borosilicate also attributed to the efficient mid-IR confinement. The invariant shape of the mode over such a broad spectrum indicates that the waveguide has low dispersion in this region, which also agrees with the optical constant displayed in Fig. 4b. When dropping various analytes onto the waveguide, the light modes revealed dissimilar spectral intensity variations for different chemicals. For the methanol wetted waveguide, the mode became a lighter spot at λ = 2.55 µm and its intensity remained bright up to λ = 2.65 µm. The increase of mode intensity was due to the formation of a top cladding layer made by the dropped methanol. Meanwhile, the mode intensity of the waveguide wetted by methanol decreased instantaneously at the longer wavelength of 2.65 µm. As for water, the mode intensity diminished as the light shifted to longer wavelengths and no waveguide mode was found beyond λ = 2.65 µm. The strong intensity attenuation observed corresponds to the -OH absorption band of water. Hence, we show that the mid-IR waveguide sensor is able to differentiate water, ethanol, and methanol since they reveal different mid-IR absorption patterns. At λ = 2.65 µm, ethanol was transparent, methanol was partially transparent, and water was fully opaque. The results are consistent with previous FTIR measurements where the -OH absorption from water increased at the shorter mid-IR regime compared to that of ethanol and methanol.

**Figure 8:**
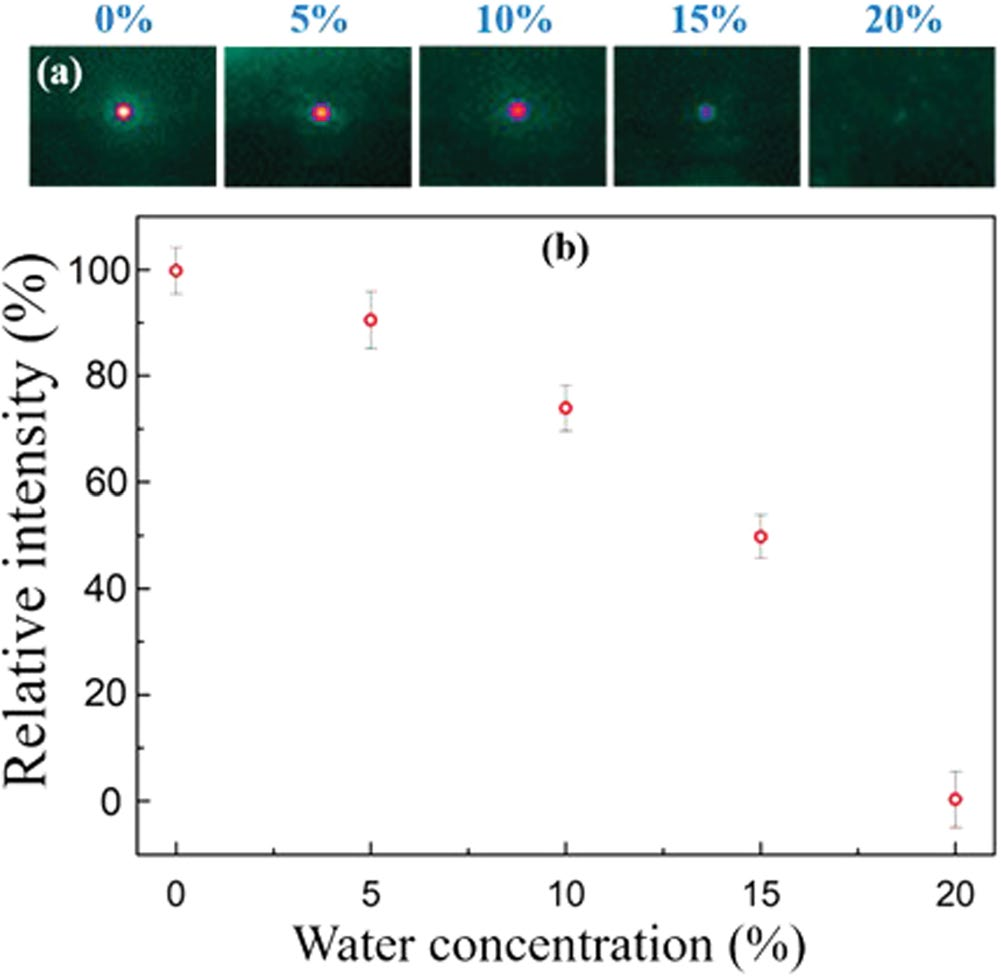
The mode image and the intensity of the AlN waveguide at λ = 2.65 µm when the waveguide was exposed to ethanol/water mixtures of different water concentrations. The mode intensity dropped promptly at higher water concentrations.

Quantitative analyses were performed by measuring water in ethanol mixtures at concentrations between 0% and 20%. The probe was set to λ = 2.65 µm light because water is absorptive at that wavelength while ethanol is transparent. As illustrated in Fig. 8, the mode intensity decreased rapidly when the concentration of water increased and eventually the mode vanished at concentration of 20%. At low concentrations, there was a 2% mode intensity variation when the water concentration changed by 1%. The water detection limit approached 1.0%. A 10% water in ethanol mixture had a 30% attenuation in the mode intensity. The waveguide light disappeared at 20% concentration showing a similar result from the simulation. Hence, the flexible mid-IR waveguide is capable of performing quantitative measurements using characteristic absorptions.

**Figure 9:**
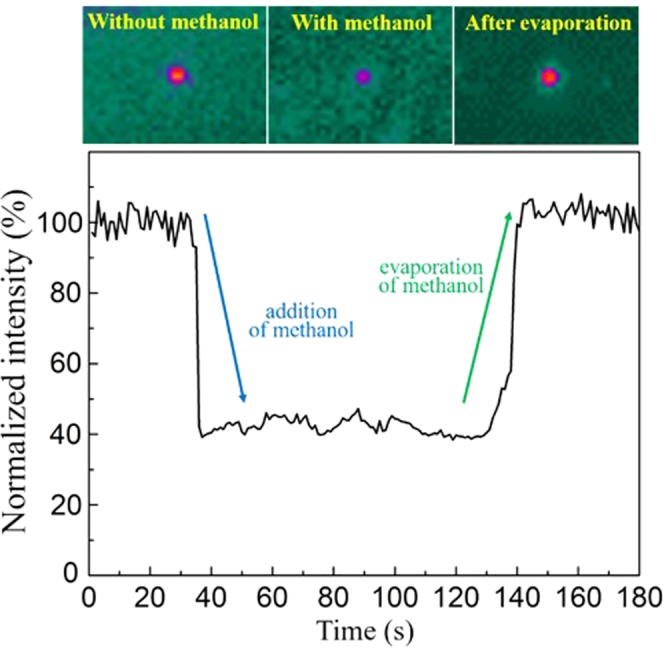
The (**a**) waveguide mode images and (**b**) transient intensity response for real-time chemical detection. Methanol is the analyte and the wavelength used was λ = 2.65 µm because it overlaps the –OH absorption of methanol. The waveguide mode intensity dropped instantaneously when the methanol wetted the waveguide surface and then recovered when the methanol evaporated.

The *in situ* sensing was then conducted by reading the waveguide mode intensity when the waveguides were exposed to the chemicals. Figure 9a illustrates the waveguide mode before and after the waveguide sensor was wetted by methanol, and Fig. 9b plots the intensity variation. The wavelength was adjusted to 2.65 µm, overlapping with the characteristic hydroxyl absorption. Before dropping methanol, the waveguide mode was clear. When methanol was dropped on the waveguide at t = 35 s, the mode instantaneously disappeared since the methanol completely absorbed the light. The waveguide mode steadily recovered after t = 130 s and eventually reached its original level because the methanol evaporated from the waveguide surface.

## Conclusions

Mid-IR flexible sensors were created by integrating AlN waveguides and an ultra-thin borosilicate template. From IR-VASE characterization, the room temperature deposited AlN film had a broad infrared transparency and low optical dispersion up to λ = 9 µm. The waveguides consisted of a 2 µm high AlN ridge structure adhered to the thin borosilicate sheet. Concentration measurements and label-free chemical identification were accomplished by waveguide mode scanning over the characteristic mid-IR absorption. The waveguide sensor can identify methanol, ethanol, and water due to the distinguishable –OH absorptions between λ = 2.50–2.65 µm. Furthermore, *in-situ* monitoring of chemicals was demonstrated. Therefore, the AlN waveguides enable a sensing device that can perform label-free and real-time chemical detection.
